# Canagliflozin Inhibits Human Endothelial Cell Proliferation and Tube Formation

**DOI:** 10.3389/fphar.2019.00362

**Published:** 2019-04-16

**Authors:** Ghazaleh Behnammanesh, Zane E. Durante, Kelly J. Peyton, Luis A. Martinez-Lemus, Scott M. Brown, Shawn B. Bender, William Durante

**Affiliations:** ^1^ Department of Medical Pharmacology and Physiology, School of Medicine, University of Missouri, Columbia, MO, United States; ^2^ Dalton Cardiovascular Research Center, University of Missouri, Columbia, MO, United States; ^3^ Research Service, Harry S. Truman Memorial Veterans Hospital, Columbia, MO, United States; ^4^ Biomedical Sciences, University of Missouri, Columbia, MO, United States

**Keywords:** canagliflozin, endothelial cells, proliferation, migration, tube formation

## Abstract

Recent clinical trials revealed that sodium-glucose co-transporter 2 (SGLT2) inhibitors significantly reduce cardiovascular events in type 2 diabetic patients, however, canagliflozin increased limb amputations, an effect not seen with other SGLT2 inhibitors. Since endothelial cell (EC) dysfunction promotes diabetes-associated vascular disease and limb ischemia, we hypothesized that canagliflozin, but not other SGLT2 inhibitors, impairs EC proliferation, migration, and angiogenesis. Treatment of human umbilical vein ECs (HUVECs) with clinically relevant concentrations of canagliflozin, but not empagliflozin or dapagliflozin, inhibited cell proliferation. In particular, 10 μM canagliflozin reduced EC proliferation by approximately 45%. The inhibition of EC growth by canagliflozin occurred in the absence of cell death and was associated with diminished DNA synthesis, cell cycle arrest, and a striking decrease in cyclin A expression. Restoration of cyclin A expression *via* adenoviral-mediated gene transfer partially rescued the proliferative response of HUVECs treated with canagliflozin. A high concentration of canagliflozin (50 μM) modestly inhibited HUVEC migration by 20%, but markedly attenuated their tube formation by 65% and EC sprouting from mouse aortas by 80%. A moderate 20% reduction in HUVEC migration was also observed with a high concentration of empagliflozin (50 μM), while neither empagliflozin nor dapagliflozin affected tube formation by HUVECs. The present study identified canagliflozin as a robust inhibitor of human EC proliferation and tube formation. The anti-proliferative action of canagliflozin occurs in the absence of cell death and is due, in part, to the blockade of cyclin A expression. Notably, these actions are not seen with empagliflozin or dapagliflozin. The ability of canagliflozin to exert these pleiotropic effects on ECs may contribute to the clinical actions of this drug.

## Introduction

Cardiovascular disease is the primary cause of morbidity and mortality in diabetes. Individuals with diabetes have a two- to four-fold increased rate of death due to cardiovascular disease relative to those without the disease, leading to a markedly diminished life span (Gu et al., 1998; Resnick and Howard, 2002; Taylor et al., 2013). Macrovascular complications of diabetes, including coronary artery disease, stroke, peripheral artery disease, and microvascular disturbances, such as retinopathy, nephropathy, and neuropathy, along with limb amputations, are responsible for much of the burden associated with this metabolic disease (Beckman et al., 2002; Fowler, 2008). Aside from its profound effect on the duration and quality of life, the increasing worldwide prevalence of diabetes will impose a severe and costly demand on health services, further emphasizing the need to develop novel therapies to manage the cardiovascular complications arising from the disease.

The pathogenesis of vascular disease in diabetes is complex and multifactorial; however, abnormalities in endothelial function play a predominant role (Creager et al., 2003; Xu and Zou, 2009). The endothelium forms the inner layer of blood vessels and is a key regulator of vascular structure and function. Besides their barrier function, endothelial cells (ECs) serve as an active signal transducer that modulates vessel wall phenotype. ECs dynamically control vascular permeability, tone, thrombosis, inflammation, and vascular smooth muscle cell proliferation and migration by synthesizing a plethora of mediators (Vane et al., 1990). Endothelial dysfunction, as represented by attenuated endothelium-dependent vasorelaxation, is a salient feature of diabetes. It has been extensively documented in animal models of diabetes and in human blood vessels from diabetic patients (Meraji et al., 1987; Durante et al., 1988; Williams et al., 1996; De Vriese et al., 2000; Rask-Madsen and King, 2007). In addition, impaired EC proliferation and migration are often detected in diabetes, and this underlies the blunted angiogenic response observed in most tissues (Santilli et al., 1992; Yan et al., 2009; Warren et al., 2014). Many of the metabolic derangements known to occur in diabetes adversely affect EC function, but hyperglycemia is believed to play a predominant role (Brownlee, 2005).

Sodium-glucose co-transporter 2 (SGLT2) inhibitors are the latest approved class of glucose-lowering drugs (Kalra, 2014). By blocking glucose uptake in the proximal tubule of the nephron, they induce glycosuria leading to decreases in both fasting and postprandial glycemia. Intriguingly, large, multicenter, clinical trials such as the EMPA-REG-OUTCOME, the CANVAS Program, and the CV-REAL Nordic have demonstrated that SGLT2 inhibitors (empagliflozin, canagliflozin, and dapagliflozin) reduce cardiovascular disease and mortality compared to other anti-hyperglycemic agents in patients with type 2 diabetes mellitus (T2DM) (Zinman et al., 2015; Birkeland et al., 2017; Neal et al., 2017). In fact, canagliflozin was recently approved by the United States Food and Drug administration for the prevention of myocardial infarction, stroke, and death among patients with type 2 diabetes, who have established cardiovascular disease. However, canagliflozin treatment was associated with a significant risk of lower limb amputations in T2DM patients, which is not seen with other SGLT2 inhibitors (Zinman et al., 2015; Birkeland et al., 2017; Fadini and Avigari, 2017; Neal et al., 2017).

The mechanisms underlying the cardiovascular benefits or amputation risk of the SGLT2 inhibitors remains unknown but they do not appear to be mediated by differential improvements in glycemic control (Zinman et al., 2015; Birkeland et al., 2017; Neal et al., 2017). While reductions in blood pressure, arterial stiffness, body weight, visceral adiposity, and/or intrarenal hemodynamics have been suggested to contribute to the cardiovascular benefits of the SGLT2 inhibitors, direct effects of these drugs on vascular cell function have not been fully considered. Given the important role that EC dysfunction plays in promoting diabetes-associated vascular disease and limb ischemia and the discrepant impact of canagliflozin on limb amputation, the present study tested the hypothesis that canagliflozin, but not other SGLT2 inhibitors, impairs EC function and angiogenesis.

## Materials and Methods

### Materials

M199 medium, streptomycin, penicillin, gelatin, heparin, trypsin, ethylenediaminetetraacetic acid (EDTA), sodium dodecyl sulfate (SDS), NaOH, elastase, cesium chloride, collagenase, phosphate-buffered saline (PBS), RNase, propidium iodide, glycerol, bromophenol blue, isolectin B4, and mercaptoethanol were from Sigma-Aldrich (St. Louis, MO). Matrigel and endothelial cell growth factor were from BD Biosciences (San Jose, CA). Antibodies against cyclin D1, cyclin E, cyclin A, p21, p27, platelet endothelial cell adhesion molecule-1 (PECAM-1), SGLT2, and β-actin were from Santa Cruz Biotechnology (Santa Cruz, CA), the antibody against SGLT1 was from GeneTex (Irvine, CA), and the antibody against phospho-retinoblastoma protein was from Cell Signaling Technologies (Beverley, MA). [^3^H]Thymidine (20 Ci/mmol) was from Perkin Elmer (Boston, MA). Canagliflozin, empagliflozin, and dapagliflozin were purchased from Selleck Chemicals (Houston, TX).

### Animals

Male C57BL/6 mice (9–10 weeks of age) were obtained from the Jackson Laboratory (Bar Harbor, ME) and housed in animal care facilities of the University of Missouri. They were maintained at ~24°C on a 12–12 h light-dark cycle with food and water available *ad libitum* for at least a week before use. For the EC isolation experiments, mice were anesthetized by an intraperitoneal injection of ketamine (87.5 mg/kg)/xylazine (12.5 mg/kg) cocktail (Butler Schein Animal Health Corporation, Dublin, OH), and aortas harvested for EC isolation and culture, as described below. For the capillary sprouting experiments, mice were anesthetized with isoflurane (2–4% in 100% O_2_) and aortas collected for the *ex vivo* experiments. This study was approved by the institutional Animal Care-Use Committees of the University of Missouri and the Truman VA Hospital (Columbia, MO, USA) and performed in accordance with the National Research Council’s Guide for the Care and Use of Laboratory Animals.

### Cell Culture

Primary human umbilical vein ECs (HUVECs) and human aortic ECs (HAECs) were purchased from Lonza Incorporated (Allendale, NJ), while primary mouse aortic ECs (MAECs) were isolated by plating mouse thoracic aortas on matrigel-coated plates and purifying MAEC outgrowth with a PECAM-1 antibody, as we have previously described (Liu et al., 2013). Primary cells were characterized as ECs by both positive staining for PECAM-1 and uptake of acetylated low-density lipoprotein by 95% of cells. Cells were serially cultured on gelatin-coated plates in M199 medium supplemented with 20% bovine calf serum, 2 mM L-glutamine, 50 μg/ml EC growth factor, 90 μg/ml heparin, and 100 U/ml of penicillin and streptomycin. All cells were incubated at 37°C in an atmosphere of 95% air and 5% CO_2_.

### Cell Proliferation and DNA Synthesis

ECs were seeded (2 × 10^4^ cells/well) onto six-well plates in serum-containing media. After 24 h, cells were washed and treated with vehicle or SGLT2 inhibitors. Media with appropriate additions were replenished every second day. Cell number determinations were performed at various times by dissociating cells with trypsin (0.05%):EDTA (0.53 mM) and counting cells using an automated cell counter (Moxi Z ORFLO Technologies, Ketchum, ID). EC proliferation was also monitored by measuring DNA synthesis (Peyton et al., 2012). Briefly, cells were pulsed with [^3^H]thymidine (1 μCi/ml [0.037 MBq]) for 4 h, washed with ice-cold PBS, fixed with 10% trichloroacetic acid for 30 min at 4°C, and DNA extracted with 0.2% SDS/0.2 N NaOH. Radioactivity was determined by scintillation counting (Tricarb liquid scintillation analyzer, model 2100, Packard, Meriden, CT).

### Cell Cycle Analysis

Cell cycle distribution was assessed in ECs grown to 70–80% confluence by flow-activated cell sorting (Peyton et al., 2012). Cells were treated in the presence and absence of SGLT2 inhibitors for 24 h. Cells were then collected, suspended in PBS, and pelleted by centrifugation at 1,000 *g* for 5 min. Pellets were washed with PBS, suspended in 70% ethanol, and fixed overnight at 4°C. Fixed cells were then incubated with propidium iodide (50 μg/ml) and RNase A (100 μg/ml) for 60 min at room temperature, and DNA fluorescence measured in a Beckman Coulter CyAN ADP Cytometer (Brea, CA). Flow cytometry results were analyzed using FlowJo^™^ software (FlowJo LLC, Ashland, OR, USA).

### Cell Migration

EC migration was determined using the scratch-wound assay (Chang et al., 2014). Confluent cell monolayers were scraped with a pipette tip to generate a wound. Cell debris was removed by several washes with PBS, and injured monolayers incubated in the presence or absence of SGLT2 inhibitors. Cell monolayers were photographed immediately and 20 h after scratch injury with a digital camera (Q-Imaging, QICAM; Hitschfel Instruments, Incorporated, St. Louis, MO), and the degree of wound closure determined by planimetry.

### Endothelial Cell Tube Formation

ECs (2 × 10^5^ cells/well) were seeded in 48-well plates that had been pre-coated with matrigel (50 μl/well). After incubation for 6 h in serum-containing media, images of tube morphology were captured by an inverted Olympus CKX41 microscope (Olympus America, Inc., Center Valley, PA), and the extent of tube formation quantified by counting the number of meshes (Khoo et al., 2011).

### Aortic Ring Capillary Sprouting Assay

Aortas were cut into 1 mm segments and then embedded and cultured in fibrinogen gels (3 mg/ml fibrinogen in Opti-MEM including 1 μl/ml thrombin) in 48 well plates, as previously described (Kiefer et al., 2004; West and Burbridge, 2009). Aortic segments were cultured in Opti-MEM (Thermofisher, Grand Island, NY) supplemented with either vascular endothelial growth factor (VEGF)-A_164_ (10 ng/ml, Invitrogen Corporation, Carlsbad, CA) alone or plus canagliflozin (10, 20, or 50 μM) for 5 days, changing the media every 2–3 days. Canagliflozin was prepared in DMSO, and accordingly, VEGF-A_164_ alone treated aortas also received 0.1% DMSO as a vehicle control. Three aortic segments from each animal received each treatment. At 5 days, segments were fixed with 3% paraformaldehyde and stained with FITC-conjugated isolectin B_4_ (Sigma-Aldrich, St. Louis, MO). Two Z-stack images of sprouting were collected from each segment *via* fluorescence confocal microscopy (Leica TCS SPE, Buffalo Grove, IL) and EC sprouting networks were reconstructed in 3D (Imaris; Bitplane AG, Zurich) to quantify total volume of sprouting networks.

### Lactate Dehydrogenase Activity Assay

Lactate dehydrogenase activity was measured using the CytoTox 96 Non-Radioactive Cytotoxicity Assay (Promega Life Sciences). Briefly, cells were seeded in 24-well plates and treated with SGLT2 inhibitors for 24 h at 37°C. After centrifugation, an aliquot of the supernatant was transferred to a 96-well plate, followed by addition of an equal amount of CytoTox 96 reagent. After 30 min of incubation at room temperature, acetic acid (1 M) was added to stop the reaction and absorbance at 490 nm measured by spectroscopy (μQuant spectrophotometer, Bio-Tek Instruments, Winooski, VT). Lactate dehydrogenase activity was reported as a percentage of control cells.

### Western Blotting

ECs were harvested in electrophoresis buffer (125 mM Tris, pH 6.8, 12.5% glycerol, 2% SDS, and trace bromophenol blue), and proteins separated by SDS-polyacrylamide gel electrophoresis. After electrophoretic transfer to nitrocellulose membranes, membranes were blocked with PBS and nonfat milk (5%), and then incubated overnight at 4°C with primary antibodies against cyclin D1 (1:200), cyclin E (1:100), cyclin A (1:500), p27 (1:250), p21 (1:250), SGLT1 (1:1,500), SGLT2 (1:100), phospho-retinoblastoma protein (1:100), or β-actin (1:200). Membranes were then washed extensively in PBS and incubated with appropriate horseradish peroxidase-labeled secondary antibodies for 60 min at room temperature. Afterward, membranes were incubated with enhanced chemoluminescence substrate for horse radish peroxidase (GE Healthcare, Chicago, IL) and the signal intensity detected by X-ray film exposure. Blots were then stripped of antibodies by incubating membranes at 50°C for 30 min with a stripping buffer (10% SDS and 100 mM mercaptoethanol, in 62.5 mM Tris buffer, pH 6.8) before probing with other primary antibodies. Protein expression was determined by densitometry and normalized with respect to β-actin (Peyton et al., 2012).

### Cyclin A Adenovirus Infection

An adenovirus expressing cyclin A (AdCyclin A) was created by cloning the human cyclin A cDNA in the pAACMVpLpA vector and co-transfecting HEK-293 cells (American Type Culture Collection, Manassas, VA) with the pJM17 plasmid (Wang et al., 2002). Virus from cytopathic HEK-293 cells was collected 48 h post infection, purified by gradient cesium chloride centrifugation, and viral titers measured by plaque assay. Subconfluent HUVECs were infected with AdCyclin A or a control adenovirus expressing the green fluorescent protein (AdGFP) at a multiplicity of infection of 50 for 24 h prior to treatment with the SGLT2 inhibitors.

### Statistical Analysis

Results are expressed as mean ± SEM of at least three independent experiments. Statistical analyses were performed with the use of a Student’s two tailed *t*-test and one way analysis of variance with the Holm-Sidak post-hoc test when more than two treatment regimens were compared. *p* less than 0.05 were considered statistically significant.

## Results

**Figure 1:**
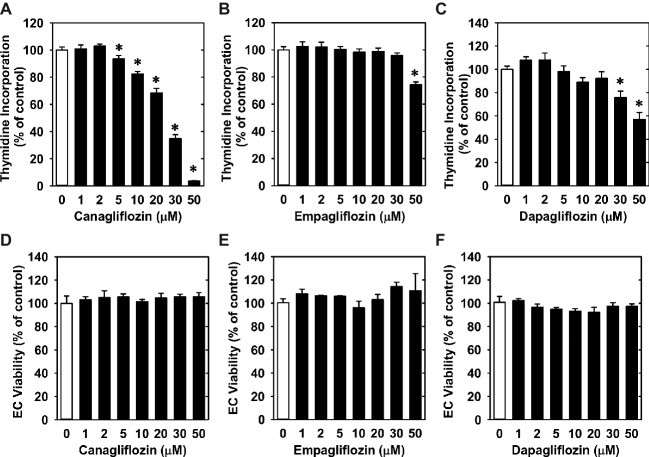
Effect of SGLT2 inhibitors on DNA synthesis and viability of HUVECs. **(A–C)** Effect of canagliflozin, empagliflozin, and dapagliflozin on DNA synthesis in HUVECs. Cells were treated with SGLT2 inhibitors (0–50 μM) for 24 h. Results are mean ± SEM (*n* = 6–8). **(D–F)** Canagliflozin, empagliflozin, and dapagliflozin do not stimulate lactate dehydrogenase activity in the culture media of HUVECs. Cells were treated with SGLT2 inhibitors (0–50 μM) for 24 h. Results are mean ± SEM (*n* = 4). *Statistically significant effect of SGLT2 inhibitors.

**Figure 2:**
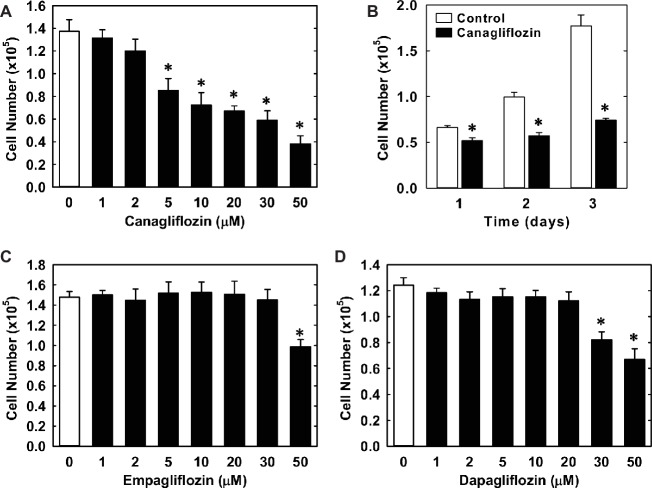
Effect of SGLT2 inhibitors on the proliferation of HUVECs. **(A)** Canagliflozin inhibits the proliferation of HUVECs in a concentration-dependent manner. Cells were treated with canagliflozin (0–50 μM) for 3 days. **(B)** Canagliflozin inhibits the proliferation of HUVECs after 1, 2, and 3 days of drug exposure. Cells were treated with canagliflozin (50 μM) for up to 3 days. **(C)** Empagliflozin inhibits the proliferation of HUVECs in a concentration-dependent manner. Cells were treated with empagliflozin (0–50 μM) for 3 days. **(D)** Dapagliflozin inhibits the proliferation of HUVECs in a concentration-dependent manner. Cells were treated with dapagliflozin (0–50 μM) for 3 days. Results are mean ± SEM (*n* = 6). *Statistically significant effect of SGLT2 inhibitors.

**Figure 3:**
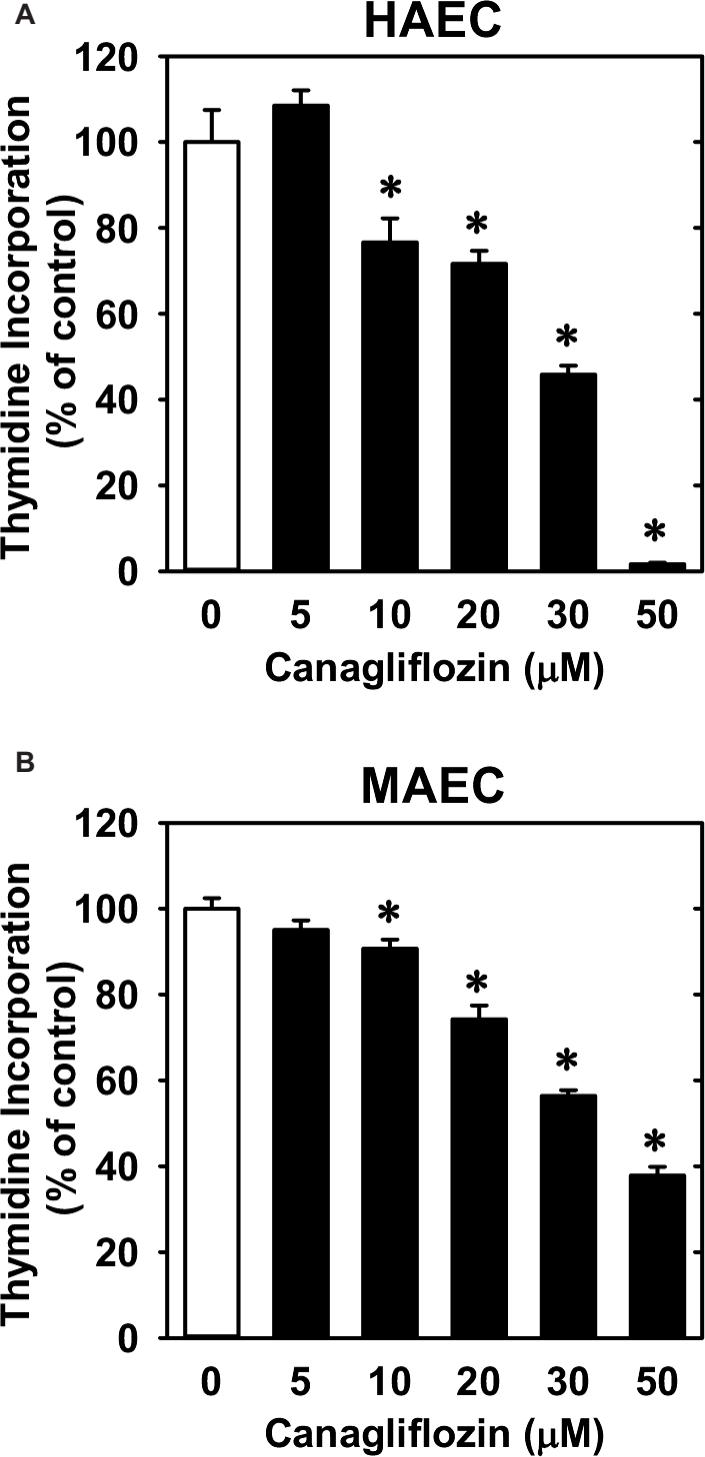
Effect of canagliflozin on DNA synthesis in HAECs and MAECs. **(A,B)** HAECs or MAECs were treated with canagliflozin (0–50 μM) for 24 h. Results are mean ± SEM (*n* = 6). *Statistically significant effect of canagliflozin.

Treatment of HUVECs with canagliflozin resulted in a concentration-dependent inhibition of DNA synthesis (Figure 1A). The ability of canagliflozin to block DNA synthesis was observed at concentrations (5–10 μM) (Figure 1A) that are achieved in the plasma of patients taking canagliflozin (Devineni et al., 2015). Moreover, DNA synthesis was nearly abolished with a higher concentration of canagliflozin (50 μM). In contrast, clinically relevant concentrations of empagliflozin and dapagliflozin (1–2 μM) (Kasichayanula et al., 2011; Brand et al., 2012) failed to inhibit DNA synthesis (Figures 1B,C). However, at very high concentrations (30–50 μM), empagliflozin and dapagliflozin modestly attenuated DNA synthesis. Notably, the inhibition of DNA synthesis by these three SGLT2 inhibitors was not related to any change in cell viability, as assessed by lactate dehydrogenase activity measurements (Figures 1D–F). In addition, incubation of HUVECs with canagliflozin induced a concentration-dependent decrease in cell proliferation, as assessed by direct cell counting (Figure 2A). A significant inhibition of cell growth by canagliflozin was noted beginning at the pharmacologically relevant concentration of 5 μM. The anti-proliferative effect of canagliflozin was detected 1 day after treatment, and this was further magnified after 3 days of canagliflozin exposure (Figure 2B). In contrast, only high concentrations (30–50 μM) of empagliflozin and dapagliflozin inhibited the proliferation of HUVEC (Figures 2C,D). Furthermore, pharmacologically relevant concentrations of canagliflozin also inhibited the proliferation of HAECs and MAECs (Figures 3A,B).

**Figure 4:**
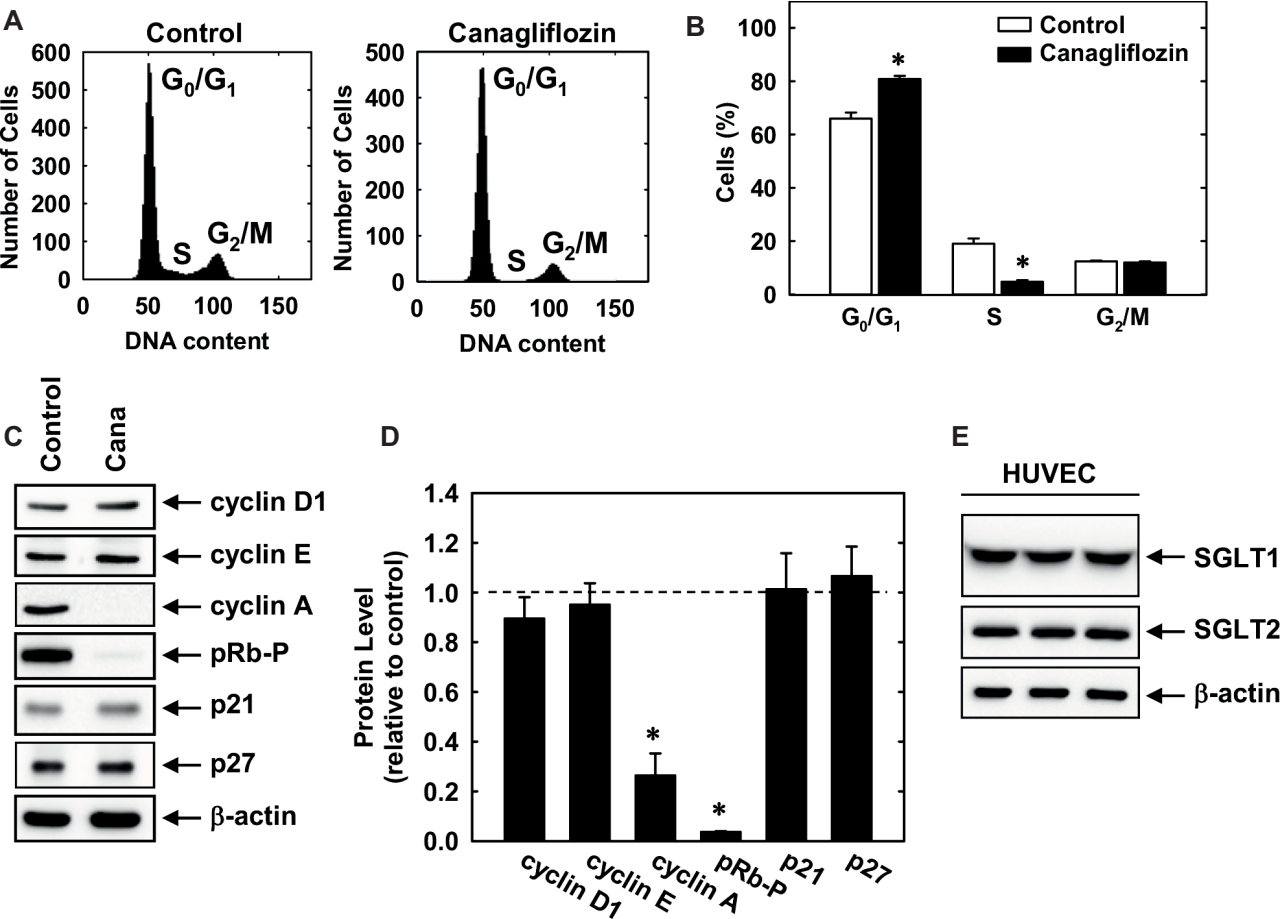
Cell cycle progression and protein expression and phosphorylation in HUVECs. **(A)** Representative histograms of HUVECs treated for 24 h in the absence or presence of canagliflozin (50 μM). **(B)** Effect of canagliflozin (50 μM) on the distribution of HUVECs in the cell cycle. **(C)** Effect of canagliflozin on the expression and phosphorylation of cell cycle regulatory proteins in HUVECs. Cells were incubated in the absence and presence of canagliflozin (50 μM) for 24 h, and protein expression and phosphorylation determined by western blotting. **(D)** Quantification of protein expression or phosphorylation relative to control cells. **(E)** HUVECs express SGLT1 and SGLT2 protein. Cell lysates from HUVEC (three independent lanes) were resolved by SDS-polyacrylamide gel electrophoresis and immunoblotted with anti-SGLT antibodies. Results are means ± SEM (*n* = 6). *Statistically significant effect of canagliflozin.

**Figure 5:**
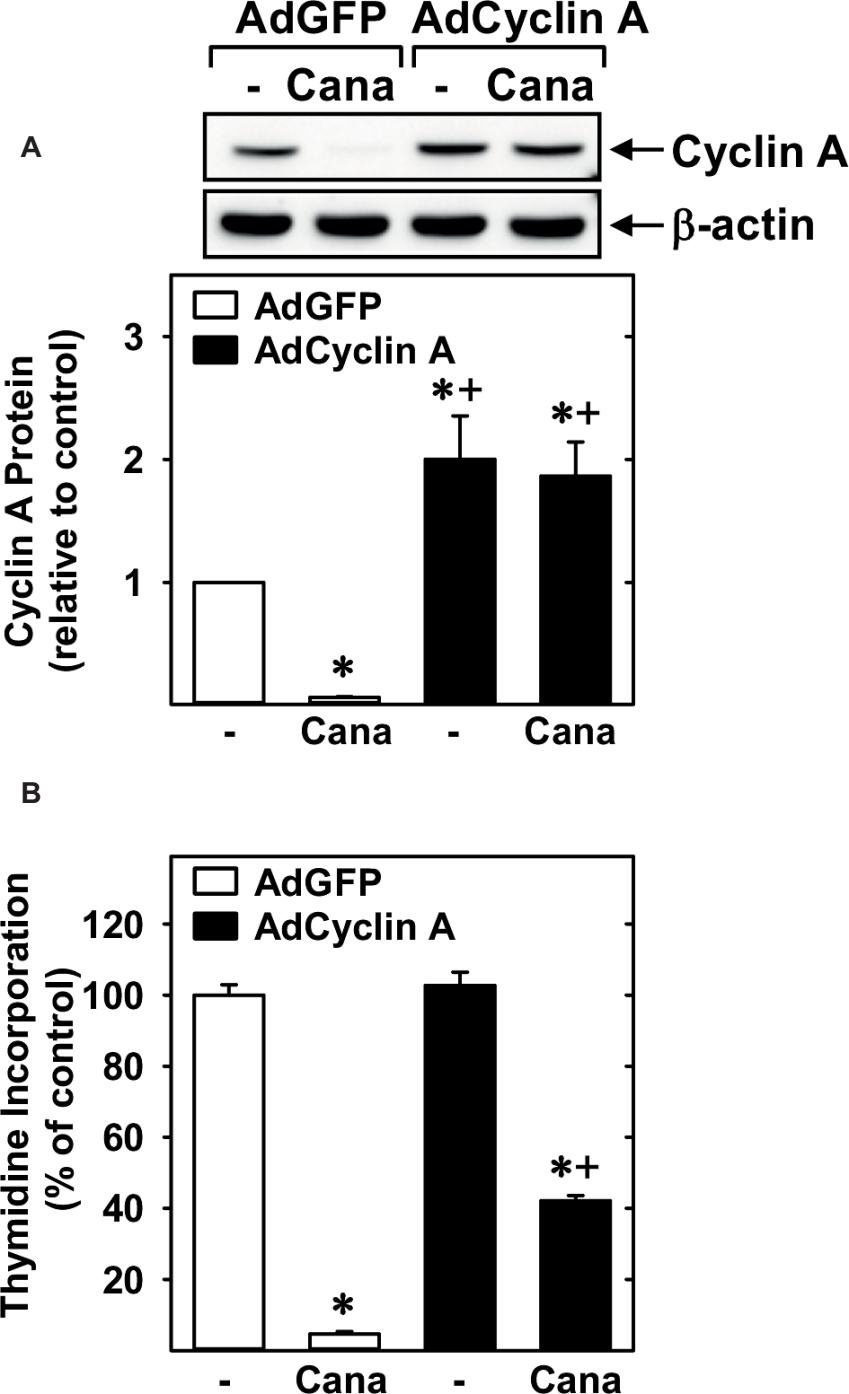
Role of cyclin A in the anti-proliferative action of canagliflozin. **(A)** AdCyclin A increases cyclin A protein levels in canagliflozin (Cana)-treated HUVECs. Results are mean ± SEM (*n* = 3). **(B)** AdCyclin A increased DNA synthesis in Cana-treated HUVECs. Cells were infected with AdCyclin A or AdGFP (50 MOI) for 24 h and then incubated in the absence or presence of Cana (50 μM) for another 24 h prior to measuring DNA synthesis. Results are mean ± SEM (*n* = 6). *Statistically significant effect of canagliflozin. ^+^Statistically significant effect of AdCyclin A.

Subsequently, we determined the effect of canagliflozin on cell cycle progression. Administration of canagliflozin-arrested HUVECs in the G_0_/G_1_ phase of the cell cycle, as demonstrated by an increase in the percentage of cells in G_0_/G_1_ with a corresponding decline in the fraction of cells in S and G_2_/M phases (Figures 4A,B). To determine the mechanism by which canagliflozin disrupts cell cycle progression, we examined the effect of canagliflozin on the expression of cell cycle regulatory proteins. Canagliflozin dramatically reduced the expression of cyclin A and the phosphorylation of retinoblastoma protein (Figures 4C,D). In contrast, canagliflozin had no significant effect on the expression of cyclin D1 and E and the cyclin-dependent kinase inhibitors p27 and p21. Western blotting also revealed the presence of both SGLT1 and SGLT2 in HUVECs (Figure 4E). Given the strong inhibition of cyclin A by canagliflozin, we tested whether restoration of cyclin A levels would negate the anti-proliferative effect of the SGLT2 inhibitor. Infection of HUVECs with AdCyclin A restored cyclin A protein expression in the presence of canagliflozin and partially rescued EC proliferation in the canagliflozin-treated cells (Figures 5A,B).

**Figure 6:**
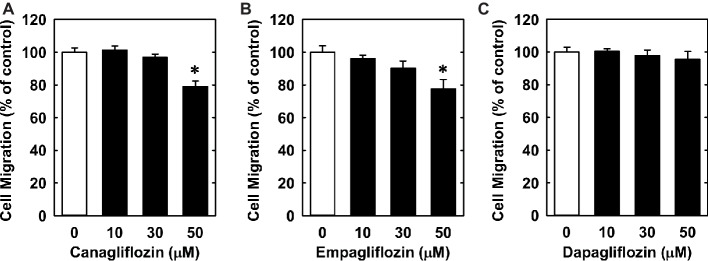
Effect of SGLT2 inhibitors on the migration of HUVECs. **(A–C)** Effect of canagliflozin, empagliflozin, and dapagliflozin on the migration of HUVECs. Results are mean ± SEM (*n* = 6). *Statistically significant effect of SGLT2 inhibitors.

**Figure 7:**
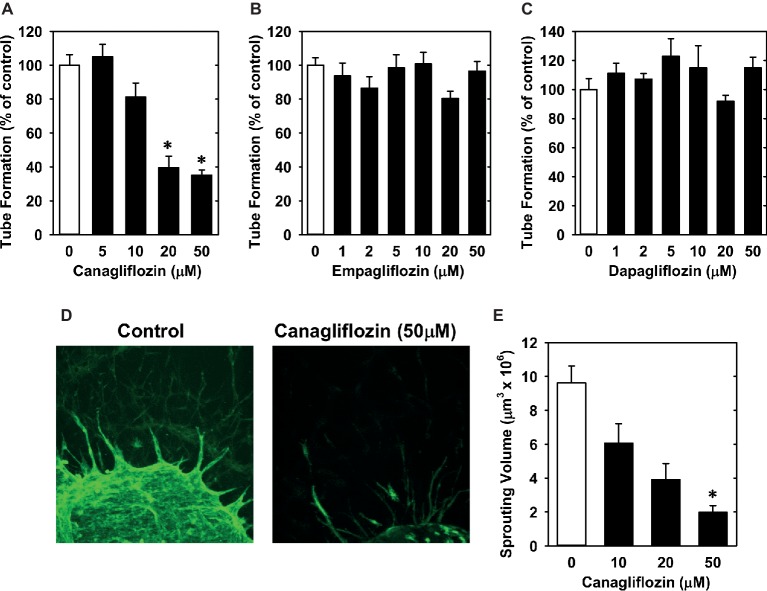
Effect of SGLT2 inhibitors on EC tube formation. **(A–C)** Effect of canagliflozin, empagliflozin, and dapagliflozin on tube formation by HUVECs. **(D)** Representative two-dimensional images of EC capillary sprouting from control or canagliflozin-treated mouse aortic rings. Aortic rings were cultured for 5 days in fibrinogen gels in the absence or presence of canagliflozin (50 μM) and EC capillary sprouting detected by fluorescence confocal microscopy following isolectin B_4_ staining. **(E)** Summary data showing the effect of canagliflozin on capillary sprouting from mouse aortic rings. Results are mean ± SEM (*n* = 4–8). *Statistically significant effect of canagliflozin.

Lastly, we investigated the effect of canagliflozin on EC migration and differentiation. While high concentrations of canagliflozin and empagliflozin (50 μM) had a modest inhibitory effect on the migration of HUVECs, dapagliflozin had no effect on EC motility (Figures 6A–C). However, canagliflozin strongly inhibited tube formation by HUVECs starting at a concentration of 20 μM (Figure 7A), whereas empagliflozin and dapagliflozin did not affect endothelial tube formation (Figures 7B,C). Finally, canagliflozin also blocked the sprouting of EC capillaries from mouse aortic rings cultured in a fibrinogen gel. Five days after culture, extensive EC capillary sprouting was observed in untreated aortas, and this was markedly attenuated by canagliflozin in a concentration-dependent fashion (Figure 7D). A downward trend in EC sprouting was noted at a concentration of 20 μM (*p* = 0.098) and a significant decrease in sprouting was observed at 50 μM (Figure 7E).

## Discussion

The present study demonstrates that canagliflozin is a robust inhibitor of EC proliferation. The anti-proliferative action of canagliflozin is observed in human ECs derived from both the venous and arterial circulation as well as ECs isolated from the murine circulation and is dependent, in part, on the suppression of cyclin A expression. This study also found that canagliflozin blocks endothelial tube formation from cultured ECs and isolated mouse aortic rings. In contrast, empagliflozin and dapagliflozin minimally affects EC proliferation and tube formation. The ability of canagliflozin to elicit these pleiotropic actions on EC function may contribute to the increased risk of limb amputation noted in diabetic patients taking this drug by impairing angiogenesis (Neal et al., 2017).

This study is the first to show that canagliflozin is an inhibitor of EC proliferation and DNA synthesis. The anti-proliferative action of canagliflozin is concentration-dependent, and significantly, is observed at concentrations (10 μM) that are achieved in the plasma of patients treated with this particular SGLT2 inhibitor (Devineni et al., 2015). The blockade of EC proliferation by canagliflozin occurs in the absence of cell death as revealed by lactate dehydrogenase activity measurements and the lack of a sub G_0_/G_1_ fraction in the cell cycle histograms, indicating that canagliflozin functions in a cytostatic rather than cytotoxic manner. The anti-proliferative action of canagliflozin appears uniform as it was observed in ECs derived from the human arterial and venous circulation as well as the mouse arterial circulation. Interestingly, the degree of inhibition is greater in human relative to mouse ECs, suggesting that human cells may be more sensitive to the growth-inhibitory action of canagliflozin. Moreover, the inhibition of EC growth by canagliflozin likely represents a compound-specific effect rather than a class effect since pharmacologically relevant concentrations of other SGLT2 inhibitors including empagliflozin and dapagliflozin (1–2 μM) (Kasichayanula et al., 2011; Brand et al., 2012) failed to block EC proliferation. Thus, this study adds to a growing list of off-targets actions elicited by canagliflozin, and further underscores the need to consider these pleiotropic effects when assessing the clinical properties of the drug (Pattanawongsa et al., 2015; Hawley et al., 2016; Villani et al., 2016; Mancini et al., 2018; Secker et al., 2018; Uthman et al., 2018).

Our finding that canagliflozin blocks EC proliferation is in agreement with earlier studies documenting robust inhibitor effects of canagliflozin on the replication of renal proximal tubule epithelial cells, lung, liver, and prostate cancer cells, and on the *in vivo* growth of xenograft tumors in athymic nude mice (Villani et al., 2016; Kaji et al., 2018; Secker et al., 2018). Notably, our results are in contrast to recent reports showing that canagliflozin has no effect on the proliferation of HUVECs (Kaji et al., 2018; Mancini et al., 2018). However, these studies examined only a single concentration of the drug over a short time period (24 and 36 h), which may be a suboptimal time interval to detect differences in the rate of cell growth. Consistent with this notion, we found that the anti-proliferative effect of canagliflozin was much greater at 72 h than after 24 h of drug exposure. Thus, longer duration experiments more closely resembling long-term use in human patients are better able to identify the anti-proliferative potential of canagliflozin in ECs.

EC proliferation involves the progression of cells through discrete stages of the cell cycle, where DNA synthesis and mitosis occur. Examination of the cell cycle distribution revealed that canagliflozin arrests ECs in the G_0_/G_1_ phase of the cell cycle and is associated with a prominent decrease in the phosphorylation of retinoblastoma protein and reduced expression of cyclin A, without altered expression of other cyclins or cyclin-dependent kinase inhibitors. The hypophosphorylation of retinoblastoma protein detected after canagliflozin treatment keeps the protein in its growth-repressive state, which curbs the transcription of genes, including cyclin A, that are required for DNA synthesis (Weinberg, 1995). Moreover, cyclin A promotes the phosphorylation and deactivation of retinoblastoma protein *via* its interaction with cyclin-dependent kinase 2, highlighting crucial interactions between these two proteins that direct cell cycle progression. Of significance, we demonstrate that adenoviral-mediated rescue of cyclin A expression in canagliflozin-treated ECs increases DNA synthesis in these cells, establishing that cyclin A is a critical target of canagliflozin. The mechanism by which canagliflozin inhibits cyclin A expression is not known. However, recent work from our laboratory and others have shown that the metabolism and entry of glutamine into the tricarboxylic acid cycle plays a fundamental role in stimulating cyclin A expression and EC proliferation (Huang et al., 2017; Kim et al., 2017; Peyton et al., 2018). Intriguingly, a recent report found that clinically relevant concentrations of canagliflozin, but not empagliflozin or dapagliflozin, prevent the replenishment of tricarboxylic acid cycle intermediates by glutamine *via* the dual inhibition of mitochondrial glutamate dehydrogenase and complex I (Secker et al., 2018), raising the possibility that canagliflozin may also retard cyclin A expression and the propagation of ECs by suppressing mitochondrial metabolism.

Notably, restoration of cyclin A does not fully restore DNA synthesis, suggesting that other factors may also contribute to the anti-proliferative action of canagliflozin. Consistent with an earlier study (Mancini et al., 2018), we found that HUVEC express SGLT2 protein. Aside from ECs, SGLT2 is found in multiple cancer cells, where it stimulates tumor growth (Kaji et al., 2018; Scafoglio et al., 2018). However, it is unlikely that inhibition of this transporter by canagliflozin mediates its anti-proliferative action in ECs as similar effects would have been observed with pharmacologically relevant concentrations of empagliflozin and dapagliflozin. Interestingly, we show for the first time that HUVECs also express SGLT1, extending previous work reporting the presence of SGLT1 in microvascular endothelial cells of the brain and heart (Elfeber et al., 2004; Sajja et al., 2014; Vrhovac et al., 2015). While canagliflozin has a relatively weak selectivity for SGLT2 over SGLT1 compared to empagliflozin and dapagliflozin (Liang et al., 2012; Kurosaki and Ogasawara, 2013), it is doubtful that canagliflozin evokes its anti-proliferative effect through SGLT1 since this glucose transporter is associated with cell survival rather than proliferation (Weihua et al., 2008). Alternatively, our laboratory has previously identified AMP-activated protein kinase (AMPK) as a potent inhibitor of EC growth (Peyton et al., 2012). Interestingly, a recent report found that clinically relevant concentrations of canagliflozin, but not empagliflozin or dapagliflozin, activate this kinase (Hawley et al., 2016), raising the possibility that AMPK may also participate in the selective growth inhibitory action of canagliflozin.

We also found that canagliflozin blocks the migration of ECs. However, a moderate decrease in EC motility was only observed at a high concentration of canagliflozin (50 μM), and this was also seen with elevated concentrations of empagliflozin but not dapagliflozin. Importantly, however, canagliflozin markedly attenuated the differentiation of ECs into tubes. This effect by canagliflozin was observed with a concentration of 20 μM and is unique to this drug as it was not detected with either empagliflozin or dapagliflozin. Furthermore, the ability of canagliflozin to block endothelial tube formation was confirmed using an *in situ* sprouting assay in mouse aortic rings. Canagliflozin tended to reduce sprouting at a concentration of 20 μM and significantly inhibited sprouting at a concentration of 50 μM, consistent with the effect of canagliflozin on EC migration. These anti-angiogenic actions occur at concentrations exceeding the typical plasma concentration in the average T2DM patient but may be particularly relevant for individuals with preexisting chronic kidney disease, a history of kidney injury, or elderly patients subjected to polypharmacy that may be exposed to much higher concentrations of canagliflozin if appropriate dose adjustments are not made (Davies et al., 2016; Fusco et al., 2016). Further, as discussed above, our data suggest that human ECs may be more sensitive than murine ECs to canagliflozin, thus, we would speculate that sprouting by human ECs may be more robustly attenuated than our results indicate using the mouse aortic ring sprouting assay.

Our finding that canagliflozin robustly inhibits EC proliferation and tube formation is of potential pharmacological significance given that these processes contribute to vascular repair and angiogenesis. Indeed, a recent report demonstrates that canagliflozin reduces intra-tumor vascularization in a xenograft model of liver cancer (Kaji et al., 2018). Thus, aside from directly inhibiting tumor cell proliferation, canagliflozin may curb tumor growth by blocking the formation of new blood vessels that are needed to nourish the growing tumor. In a similar fashion, canagliflozin may be of benefit in patients with proliferative diabetic retinopathy, where unrestrained EC proliferation and angiogenesis contributes to the disease process (Simó et al., 2006). Moreover, since angiogenesis contributes to atherosclerotic plaque progression and vulnerability (Virmani et al., 2005; Camere et al., 2017), canagliflozin may also mitigate the increased burden of atherosclerosis and its clinical complications in diabetes. In support of this concept, canagliflozin has recently been demonstrated to attenuate the progression of atherosclerosis in atherogenic mice (Nasiri-Ansari et al., 2018). On a cautionary note, by impairing the proliferation and differentiation of ECs, canagliflozin may further minimize limb blood flow in T2DM patients suffering from peripheral arterial disease. Surprisingly, a recent report found that canagliflozin accelerates the recovery of hind limb blood flow following femoral artery ligation and excision in diabetic mice, suggesting that the increased risk of amputation reported with canagliflozin may not be related to an increase in limb ischemia (Sherman et al., 2018). However, this preclinical animal model does not fully mimic the clinical setting where chronic limb ischemia in T2DM patients arises from progressive and pervasive atherosclerosis. Finally, canagliflozin may negatively impact clinical outcomes following coronary angioplasty by impairing the re-endothelialization of injured arteries, which is dependent, in part, on the proliferation of ECs from the injured border zone or from branching vessels adjacent to the site of injury (Hagensen et al., 2012). Given these latter concerns, restricting canagliflozin use, or switching to other anti-hyperglycemic medications seems advisable in patients with peripheral artery disease or in patients undergoing coronary artery stenting.

Finally, our studies were done in cultured human ECs and isolated mouse arteries, which do not fully mimic the complex biochemical and biophysical interactions that occur *in vivo*. Although extension of our *in vitro* and *ex vivo* experiments to an intact animal is important, our finding that human ECs are more sensitive to the anti-proliferative action of canagliflozin than murine ECs, suggest that studies in rodents may not accurately reflect what occurs in humans. Another limitation in our study is that the mechanism by which canagliflozin blocks EC growth has not been fully disclosed. While inhibition of cyclin A expression contributes to the anti-proliferative effect of canagliflozin, other unidentified mediators are also involved. Moreover, our study detected the presence of both SGLT1 and SGLT2 in ECs. Given that a majority of active glucose uptake by ECs is mediated by the GLUT/SLC2A family of transporters (Tumova et al., 2016), the functional significance of SGLT1/2 in these cells is uncertain and will require additional studies.

In conclusion, the present study identified canagliflozin as a potent inhibitor of human EC proliferation. The anti-proliferative action of canagliflozin is observed in ECs isolated from both the venous and arterial circulation, and is partly due to the blockade of cyclin A expression. In addition, this study found that canagliflozin inhibits tube formation in cultured ECs and mouse aortic rings. Notably, these actions are specific for canagliflozin and not seen with other SGLT2 inhibitors. The ability of canagliflozin to exert these pleiotropic effects on EC function may contribute to both the adverse and salutary actions of this drug on cardiovascular function in T2DM patients.

## Ethics Statement

This study was approved by the institutional Animal Care-Use Committees of the University of Missouri and the Truman VA Hospital (Columbia, MO, USA) and performed in accordance with the National Research Council’s Guide 152 for the Care and Use of Laboratory Animal.

## Author Contributions

GB, SMB, SBB, KP, LM-L, and WD participated in research design. GB, SMB, ZD, and KP conducted experiments and performed data analysis. GB, SMB, SBB, LM-L, KP, and WD wrote or contributed to writing of the manuscript.

### Conflict of Interest Statement

The authors declare that the research was conducted in the absence of any commercial or financial relationships that could be construed as a potential conflict of interest.

## References

[ref1] Beckman J. A. Creager M. A. Libby P. (2002). Diabetes and atherosclerosis: epidemiology, pathophysiology, and management. JAMA. 287, 2570–2581. 10.1001/jama.287.19.2570, PMID: 12020339

[ref2] Birkeland K. I. Jørgensen M. E. Carstensen B. Persson F. Gulseth H. L. Thuresson M.. (2017). Cardiovascular mortality and morbidity in patients with type 2 diabetes following initiation of sodium-glucose co-transporter-2 inhibitors versus other glucose-lowering drugs (CVD-REAL Nordic): a multinational observational analysis. Lancet Diabetes Endocrinol. 5, 709–717. 10.1016/S2213-8587(17)30258-9, PMID: 28781064

[ref3] Brand T. Macha S. Mattheus M. Pinnetti S. Woerle H. J. (2012). Pharmacokinetics of empagliflozin, a sodium glucose cotransporter-2 (SGLT-2) inhibitor, coadministered with sitagliptin in healthy volunteers. Adv. Ther. 29, 889–899. 10.1007/s12325-012-0055-3, PMID: 23054692

[ref4] Brownlee M. (2005). The pathobiology of diabetic complications. Diabetes 54, 1615–1625. 10.2337/diabetes.54.6.1615, PMID: 15919781

[ref5] Camere C. Pucelle M. Negre-Salvayre A. Salvayre R. (2017). Angiogenesis in atherosclerotic plaque. Redox Biol. 12, 18–34. 10.1016/j.redox.2017.01.007, PMID: 28212521 PMC5312547

[ref6] Chang C. F. Liu X. M. Peyton K. J. Durante W. (2014). Heme oxygenase-1 counteracts contrast media-induced endothelial dysfunction. Biochem. Pharmacol. 87, 303–311. 10.1016/j.bcp.2013.11.002, PMID: 24239896 PMC3947226

[ref7] Creager M. A. Luscher T. F. Cosentino F. Beckman J. A. (2003). Diabetes and vascular disease. Pathophysiology, clinical consequences, and medical therapy: part I. Circulation 108, 1527–1532. 10.1161/01.CIR.0000091257.27563.32, PMID: 14504252

[ref8] Davies M. Chatterjee S. Khunti K. (2016). The treatment of type 2 diabetes in the presence of renal impairment: what should we know about the newer therapies. Clin. Pharm. 8, 61–81. 10.2147/CPAA.S82008, PMID: 27382338 PMC4922775

[ref9] De Vriese A. S. Verbeuren T. J. Van de Voorde J. Lameire N. H. Vanhoutte P. M. (2000). Endothelial dysfunction in diabetes. Br. J. Pharmacol. 130, 963–974. 10.1038/sj.bjp.0703393, PMID: 10882379 PMC1572156

[ref10] Devineni D. Polidori D. Curtin C. R. Murphy J. Wang S.-S. Stieltjes H.. (2015). Pharmacokinetics and pharmacodynamics of once-and twice-daily multiple-doses of canagliflozin, a selective inhibitor of sodium glucose co-transporter 2, in healthy participants. Int. J. Clin. Pharmacol. Ther. 53, 438–446. 10.5414/CP202324, PMID: 25907176

[ref11] Durante W. Sen A. K. Sunahara F. A. (1988). Impairment of endothelium-dependent relaxation in aortae from spontaneously diabetic rats. Br. J. Pharmacol. 94, 463–468. 10.1111/j.1476-5381.1988.tb11548.x, PMID: 3134969 PMC1854002

[ref12] Elfeber K. Kohler A. Lutzenburg M. Osswald C. Galla H. J. Witte O. W.. (2004). Localization of the Na+− D-glucose cotransporter SGLT1 in the blood-brain barrier. Histochem. Cell Biol. 121, 201–207. 10.1007/s00418-004-0633-9, PMID: 14986005

[ref13] Fadini G. P. Avigari A. (2017). SGLT2 inhibitors and amputations in the US FDA adverse event reporting system. Lancet Diabetes Endocrinol. 5, 680–681. 10.1016/S2213-8587(17)30257-7, PMID: 28733172

[ref14] Fowler M. J. (2008). Microvascular and macrovascular complications of diabetes. Clin. Diabetes 26, 77–82. 10.2337/diaclin.26.2.77

[ref15] Fusco S. Garasco S. Corsonello A. Vena S. Mari V. Gareri P.. (2016). Medication-induced nephrotoxicity in older patients. Curr. Drug Metab. 17, 608–625. 10.2174/1389200217666160406115959, PMID: 27048182

[ref16] Gu K. Crowe C. C. Harris M. I. (1998). Mortality in adults with and without diabetes in a national cohort of the U.S. population, 1971–1993. Diabetes Care 21, 1138–1145. 10.2337/diacare.21.7.1138, PMID: 9653609

[ref17] Hagensen M. K. Raarup M. K. Mortensen M. B. Thim T. Nyengaard J. R. Falk E.. (2012). Circulating endothelial progenitor cells do not contribute to regeneration of endothelium after murine injury. Cardiovasc. Res. 93, 223–231. 10.1093/cvr/cvr278, PMID: 22012957

[ref18] Hawley S. A. Ford R. J. Smith B. K. Gowans G. J. Mancini S. J. Pitt R. D.. (2016). The Na^+^/glucose cotransporter inhibitor canagliflozin activates AMPK by inhibiting mitochondrial function and increasing cellular AMP levels. Diabetes 65, 2784–2794. 10.2337/db16-0058, PMID: 27381369 PMC5689380

[ref19] Huang H. Vandekeere S. Kalucka J. Bierhansl L. Zecchin A. Bruning U.. (2017). Role of glutamine and interlinked asparagine metabolism in vessel formation. EMBO J. 36, 2334–2352. 10.15252/embj.201695518, PMID: 28659375 PMC5556263

[ref20] Kaji K. Nishimura N. Seki K. Sato S. Saikawa S. Nakanishi K.. (2018). Sodium glucose cotransporter 2 inhibitor canagliflozin attenuates liver cancer cell growth and angiogenic activity by inhibiting glucose uptake. Int. J. Cancer 142, 1712–1722. 10.1002/ijc.31193, PMID: 29205334

[ref21] Kalra S. (2014). Sodium glucose co-transporter 2 (SGLT2) inhibitors: a review of their basic and clinical pharmacology. Diabetes Ther. 5, 355–366. 10.1007/s13300-014-0089-4, PMID: 25424969 PMC4269649

[ref22] Kasichayanula S. Chang M. Hasegawa M. Liu X. Yamahira N. LaCreta F. P.. (2011). Pharmacokinetics and pharmacodynamics of dapagliflozin, a novel selective inhibitor of sodium–glucose co-transporter type 2, in Japanese subjects without and with type 2 diabetes mellitus. Diabetes Obes. Metab. 13, 357–365. 10.1111/j.1463-1326.2011.01359.x, PMID: 21226818

[ref23] Khoo C. P. Micklem K. Watt S. M. (2011). A comparison of methods for quantifying angiogenesis in the Matrigel assay in vitro. Tissue Eng. Part C. Methods 17, 895–906. 10.1089/ten.tec.2011.0150, PMID: 21517696 PMC3162381

[ref24] Kiefer F. N. Munk V. C. Humar R. Dieterle T. Landmann L. Battegay E. J. (2004). A versatile in vitro assay for investigating angiogenesis of the heart. Exp. Cell Res. 300, 272–282. 10.1016/j.yexcr.2004.06.032, PMID: 15474993

[ref25] Kim B. Li J. Jang C. Arany Z. (2017). Glutamine fuels proliferation but not migration of endothelial cells. EMBO J. 36, 2321–2333. 10.15252/embj.201796436 28659379 PMC5556269

[ref26] Kurosaki E. Ogasawara H. (2013). Ipragliflozin and other sodium-glucose cotransporter-2 (SGLT2) inhibitors in the treatment of type 2 diabetes: preclinical and clinical data. Pharmacol. Ther. 139, 51–59. 10.1016/j.pharmthera.2013.04.003, PMID: 23563279

[ref27] Liang Y. Arakawa K. Ueta K. Matsushita Y. Kuriyama C. Martin T.. (2012). Effect of canagliflozin on renal threshold for glucose, glycemia, and body weight in normal and diabetic animal models. PLoS One 7:e30555. 10.1371/journal.pone.0052269, PMID: 22355316 PMC3280264

[ref28] Liu X. M. Peyton K. J. Durante W. (2013). Physiological cyclic strain promotes endothelial cell survival via the induction of heme oxygenase-1. Am. J. Physiol. Heart Circ. Physiol. 304, H1634–H1643. 10.1152/ajpheart.00872.2012, PMID: 23604711 PMC3680772

[ref29] Mancini S. J. Boyd D. Katwan O. J. Strembitska A. Almabrouk T. A. Kennedy S. (2018). Canagliflozin inhibits interleukin-1β-stimulated cytokine and chemokine secretion in vascular endothelial cells by AMPK-activated protein kinase-depenent and independent mechanisms. Sci. Rep. 8:5276. 10.1038/s41598-018-23420-4 29588466 PMC5869674

[ref30] Meraji S. Jayakody L. Senaratne M. P. Thomson A. B. Kappagoda T. (1987). Endothelium-dependent relaxation in aorta of BB rat. Diabetes 36, 978–981. 10.2337/diab.36.8.978, PMID: 3596063

[ref31] Nasiri-Ansari N. Dimitriadis G. K. Agrogiannis G. Perrea D. Kostakis I. D. Kaltsas G. (2018). Canagliflozin attenutates the progression of atherosclerosis and inflammation process in APOE knockout mice. *Cardiovasc. Diabetol*. 17:106. 10.1186/s12933-018-0749-1 30049285 PMC6063004

[ref32] Neal B. Perkovic V. Mahaffey K. W. de Zeeuw D. Fulcher G. Erondu N.. (2017). Canagliflozin and cardiovascular and renal events in type 2 diabetes. *N. Engl. J*. *Med.* 377, 644–657. 10.1056/NEJMoa1611925, PMID: 28605608

[ref33] Pattanawongsa A. Chau N. Rowland A. Miners J. O. (2015). Inhibition of human UDP-glucuronosyltransferase enzymes by canagliflozin and dapagliflozin: implications for drug-drug interactions. Drug Metab. Dispos. 43, 1468–1476. 10.1124/dmd.115.065870, PMID: 26180128

[ref34] Peyton K. J. Liu X. M. Yu Y. Yates B. Behnammanesh G. Durante W. (2018). Glutaminase-1 stimulates the proliferation, migration, and survival of human endothelial cells. Biochem. Pharmacol. 156, 204–214. 10.1016/j.bcp.2018.08.032, PMID: 30144404 PMC6248344

[ref35] Peyton K. J. Liu X. M. Yu Y. Yates B. Durante W. (2012). Activation of AMP-activated protein kinase inhibits the proliferation of human endothelial cells. J. Pharmacol. Exp. Ther. 342, 827–834. 10.1124/jpet.112.194712, PMID: 22700432 PMC3422516

[ref36] Rask-Madsen C. King G. L. (2007). Mechanisms of disease: endothelial dysfunction in insulin resistance and diabetes. Nat. Clin. Pract. Endocrinol. Metab. 3:46–56. 10.1038/ncpendmet0366, PMID: 17179929

[ref37] Resnick H. E. Howard B. V. (2002). Diabetes and cardiovascular disease. Annu. Rev. Med. 53, 245–267. 10.1146/annurev.med.53.082901.103904, PMID: 11818473

[ref38] Sajja R. K. Prasad S. Cucullo L. (2014). Impact of altered glycemia on blood-brain barrier endothelium: an in vitro study using the hCMEC/D3 cell line. Fluids Barrier CNS 11:8. 10.1186/2045-8118-11-8, PMID: 24708805 PMC3985548

[ref39] Santilli S. Fiegel V. Aldridge D. Knighton D. (1992). The effect of diabetes on the proliferation of aortic endothelial cells. Ann. Vasc. Surg. 6, 503–510. 10.1007/BF02000821, PMID: 1463663

[ref40] Scafoglio C. R. Villegas B. Abdelhady G. Bailey S. T. Liu J. Shirali A. S.. (2018). Sodium glucose transporter 2 is a diagnostic and therapeutic target for early stage lung adenocarcinoma. *Sci. Transl. Med*. 10:eaat5933. 10.1126/scitranslmed.aat5933, PMID: 30429355 PMC6428683

[ref41] Secker P. F. Beneke S. Schlichenmaier N. Delp J. Gutbier S. Leist M. (2018). Canagliflozin mediated dual inhibition of mitochondrial glutamate dehydrogenase and complex I: an off-target adverse effect. Cell Death Dis. 9:226. 10.1038/s41419-018-0273-y 29445145 PMC5833677

[ref42] Sherman S. E. Bell G. I. Teoh H. Al-Omran M. Connelly K. A. Bhatt D. L. (2018). Canagliflozin improves the recovery of blood flow in an experimental model of severe limb ischemia. JCC Basic Trans. Sci. 3, 327–329. 10.1016/j.jacbts.2018.01.010 PMC6059819 30062217

[ref43] Simó R. Carrasco E. García-Ramírez M. Hernández C. (2006). Angiogenic and antiangiogenic factors in proliferative diabetic retinopathy. Curr. Diabetes Rev. 2, 71–98. 10.2174/157339906775473671, PMID: 18220619

[ref44] Taylor K. S. Heneghan C. J. Farmer A. J. Fuller A. M. Adler A. I. Aronson J. K.. (2013). All-cause and cardiovascular mortality in middle-aged people with type 2 diabetes compared with people without diabetes in a large U.K. primary care database. Diabetes Care 36, 2366–2371. 10.2337/dc12-1513, PMID: 23435157 PMC3714501

[ref45] Tumova S. Kerimi A. Porter K. E. Williamson G. (2016). Transendothelial glucose transport is not restricted by extracellular hyperglycaemia. *Vasc. Pharmacol*. 87, 219–229. 10.1016/j.vph.2016.11.001, PMID: 27825869

[ref46] Uthman L. Baartscheer A. Bleijlevens B. Schumacher C. A. Fiolet J. W. T. Koeman A. (2018). Class effects of SGLT2 inhibitors in mouse cardiomyocytes and hearts: inhibition of Na+/H+ exchanger, lowering cytosolic Na+ and vasodilation. Diabetologia 61, 722–726. 10.1007/s00125-017-4509-7, PMID: 29197997 PMC6448958

[ref47] Vane J. R. Änggård E. E. Botting R. M. (1990). Regulatory functions of the vascular endothelium. N. Engl. J. Med. 323, 27–36. 10.1056/NEJM199007053230106, PMID: 2113184

[ref48] Villani L. A. Smith B. K. Marcinko K. Ford R. J. Broadfield L. A. Green A. E.. (2016). The diabetes medication canagliflozin reduces cancer cell proliferation by inhibiting mitochondrial complex-I supported respiration. Mol. Metab. 5, 1048–1056. 10.1016/j.molmet.2016.08.014, PMID: 27689018 PMC5034684

[ref49] Virmani R. Kolodgie F. D. Burke A. P. Finn A. V. Gold H. K. Tulenko T. N.. (2005). Atherosclerotic plaque progression and vulnerability to rupture: angiogenesis as a source of intraplaque hemorrhage. Arterioscler. Thromb. Vasc. Biol. 25, 2054–2061. 10.1161/01.ATV.0000178991.71605.18, PMID: 16037567

[ref50] Vrhovac I. Balen Eror D. Klessen D. Burger C. Greljak D. Kraus O.. (2015). Localizations of Na(+)-D-glucose cotransporter SGLT1 and SGLT2 in human kidney and of SGLT1 in human small intestine, liver, lung, and heart. Pflugers Arch. 467, 1881–1898. 10.1007/s00424-014-1619-7, PMID: 25304002

[ref51] Wang H. Jiang X. Yang F. Chapman G. B. Durante W. Sibinga N. E.. (2002). Cyclin A transcriptional suppression is the major mechanism mediating homocysteine-induced endothelial cell growth inhibition. Blood 99, 939–945. 10.1182/blood.V99.3.939, PMID: 11806997 PMC5539868

[ref52] Warren C. M. Ziyad S. Briot A. Der A. Iruela-Arispe M. L. (2014). A ligand-independent VEGFR2 signaling pathway limits angiogenic responses in diabetes. Sci. Signal. 7, ra1. 10.1126/scisignal.2004235, PMID: 24399295 PMC4030697

[ref53] Weihua Z. Tsan R. Huang W. C. Wu Q. Chiui C. H. Fidler I. J. (2008). Survival of cancer cells is maintained by EGFR independent of its kinase activity. Cancer Cell 13, 385–393. 10.1016/j.ccr.2008.03.015, PMID: 18455122 PMC2413063

[ref54] Weinberg R. A. (1995). The retinoblastoma protein and cell cycle control. Cell 81, 323–330. 10.1016/0092-8674(95)90385-2, PMID: 7736585

[ref55] West D. C. Burbridge M. F. (2009). “Three-dimensional in-vitro angiogenesis in the rat aortic ring model” in Angiogenesis protocols. 2nd Edn. eds. Murray C. Martin S. (New Jersey: Humana Press), 189–210. 10.1007/978-1-59745-241-0_11 19301672

[ref56] Williams S. B. Cusco J. A. Roddy M.-A. Johnstone M. T. Creager M. A. (1996). Impaired nitric oxide-mediated vasodilation in patients with non-insulin-dependent diabetes mellitus. J. Am. Coll. Cardiol. 27, 567–574. 8606266 10.1016/0735-1097(95)00522-6

[ref57] Xu J. Zou M. H. (2009). Molecular insights and therapeutic targets for diabetic endothelial dysfunction. Circulation 120, 1266–1286. 10.1161/CIRCULATIONAHA.108.835223, PMID: 19786641 PMC2910587

[ref58] Yan J. Tie G. Park B. Yan Y. Nowicki P. T. Messina L. M. (2009). Recovery from hind limb ischemia is less effective in type 2 than in type 1 diabetic mice: roles of endothelial nitric oxide synthase and endothelial progenitor cells. J. Vasc. Surg. 50, 1412–1422. 10.1016/j.jvs.2009.08.007, PMID: 19837544 PMC2797079

[ref59] Zinman B. Wanner C. Lachin J. M. Fitchett D. Bluhmki E. Hantel S. (2015). Empagliflozin, cardiovascular outcomes, and mortality in type 2 diabetes. N. Engl. J. Med. 373, 2117–2128. 10.1056/NEJMoa1504720 26378978

